# Supplementary material for: The Dynamic of Polyphenols Concentrations in Organic and Conventional Sour Cherry Fruits: Results of a 4-Year Field Study
Source: Molecules. 2020 Aug 15;25(16):3729. doi: 10.3390/molecules25163729 (PMC7464618; doi:10.3390/molecules25163729)
Supplement: Supplementary file 1 [file molecules-25-03729-s001.pdf]

Table S1, The data about soil and orchards conditions as well fertilization and plant protection in time of sour cherry cultivation in four years of experiment

	Organic orchard	Conventional orchard
type of soil	podsolic soil overlying clay	podsolic soil overlying clay
% humus content	1.36%	1.72%
pH	6.70	6.45
fertilization (dose in kg or t/ha)	Tables 2A-3A	Tables 4A-5A
spacing of trees	4.2 x 2.5 m	4.2 x 2.5 m
plant protection	Table 3A and 5A	Table 2A and 4A

Table S2 Fertilization regime in conventional orchard in experimental time 2016-2018

**Data**	**kind of fertilizer**	**dose of fertilizer**
18 of May 2016	Krista Leaf Fruit controller	3 kg/ha
	Magnesium sulphate	1% (600 l/ha)
	Urea	0.5% (600 l/ha)
3 of June 2016	Urea	0.5% (600 l/ha)
28 of April 2017	Amonium nitrate	100 lg/ha
19 of May 2017	Magnessium sulphante	1% (600 l/ha)
	Urea	0.5% (600 l/ha)
5 of November 2017	Radkovit calcium carbonate	1.5 t/ha
18 of April 2018	Urea	0.5% (600 l/ha)
19 of April 2018	Ammonium nitrate	100 kg/ha
9 of May 2018	Magnessium sulphate	1% (600 l/ha)
	Urea	0.5% (600 l/ha)
21 of May 2018	Urea	0.5% (600 l/ha)
24 of May 2018	Urea	0.5% (600 l/ha)

Table S3 Fertilization regime in organic orchard in experimental time 2016-2018

Data	kind of fertilizer	dose of fertilizer
5 of May-14 of June 2016	cow slurry	10 m^3/ha
25 of May 2017	cow slurry	10 m^3/ha
4-17 of April 2018	cow manure	30 t/ha
20 of April 2018	Efective Microorganisms (Farma Plus)	20 l/ha

Table S4 Plant protection regime in conventional orchard in experimental time 2015-2018

Data	kind of pesticide	dose of pesticide	reason
19 of April 2015	Rovlar aquaflo 500 SC	1.25 l/ha	brown rot
25 of May 2015	Syllit 65 WP	1.5 kg/ha	leaf spot of stone trees
14 of June 2015	Merpan 80 WG	1.9 kg/ha	leaf spot of stone trees, bitter rot
28 of June 2015	Mospilan 20 SP	0.2 ka/ha	cherry fruit fly, aphids
15 of July 2015	Kaptan Plus 71.5 WP	2 kg/ha	brown rot, leaf spot of stone trees, bitter rot
23 of April 2016	Rovlar aquaflo 500 SC	1.25 l/ha	brown rot
18 of May 2016	Syllit 65 WP	1.5 kg/ha	leaf spot of stone trees
3 of June 2016	Merpan 80 WG	1.9 kg/ha	leaf spot of stone trees, bitter rot
	Mospilan 20 SP	0.2 ka/ha	cherry fruit fly, aphids
27 of June 2016	Kaptan Plus 71.5 WP	2 kg/ha	brown rot, leaf spot of stone trees, bitter rot
28 of March 2017	Cuprate Extra 350 SC	3 l/ha	brown rot, leaf spot of stone trees, bitter rot
29 of April 2017	Rovlar aquaflo 500 SC	1.25 l/ha	brown rot
19 of May 2017	Syllit 65 WP	1.2 kg/ha	leaf spot of stone trees
	Calypso	0.2 l/ha	cherry fruit fly, aphids
9 of June 2017	Kaptan Plus	2 kg/ha	brown rot, leaf spot of stone trees, bitter rot
	Decis Mega	0.25 l/ha	cherry fruit fly, aphids
22 of June 2017	Merpan 80 WG	1.9 kg/ha	leaf spot of stone trees, bitter rot
	Decis Mega	0.25 l/ha	cherry fruit fly, aphids
3 of August 2017	Syllit 65 WP	1.2 kg/ha	leaf spot of stone trees
7 of November 2017	Cuprate Extra 350 SC	3 l/ha	brown rot, leaf spot of stone trees, bitter rot
21 of November 2017	Cuprate Extra 350 SC	3 l/ha	brown rot, leaf spot of stone trees, bitter rot
11 of April 2018	Cuprate Extra 350 SC	3 l/ha	brown rot, leaf spot of stone trees, bitter rot
18 of April 2018	Signum	0.75 kg/ha	brown rot
9 of May 2018	Syllit 65 WP	1.2 kg/ha	leaf spot of stone trees
	Teppeki	0.14 kg/ha	aphids
21 of May 2018	Kaptan Plus	2 kg/ha	leaf spot of stone trees, bitter rot
24 of May 2018	Calypso	0.2 l/ha	cherry fruit fly, aphids
30 of May 2018	Switch	0.6 kg/ha	brown rot
	Decis Mega	0.25 l/ha	cherry fruit fly, aphids
8 of June 2018	Decis Mega	0.25 l/ha	cherry fruit fly, aphids
14 of August 2018	Syllit 65 WP	1.5 kg/ha	leaf spot of stone trees
7 of November 2018	Cuprate Extra 350 SC	3 l/ha	brown rot, leaf spot of stone trees, bitter rot

Table S5 Plant protection regime in conventional orchard in experimental time 2015-2018

Data	kind of pesticide	dose of pesticide	reason
20 of April 2015	Cuprate Extra 350 SC	3 l/ha	brown rot, leaf spot of stone trees, bitter rot
10 of June 2015	garden soap K + methylated spirits	15 l/ha + 15 l/ha	aphids
15 of June 2015	garden soap K + methylated spirits	15 l/ha + 15 l/ha	aphids
10 of July 2015	garden soap K + methylated spirits	15 l/ha + 15 l/ha	aphids
16 of July 2015	garden soap K + methylated spirits	15 l/ha + 15 l/ha	aphids
21 of July 2015	garden soap K + methylated spirits	15 l/ha + 15 l/ha	aphids
9 of August 2015	garden soap K + methylated spirits	15 l/ha + 15 l/ha	aphids
23 of April 2016	Cuprate Extra 350 SC	3 l/ha	brown rot, leaf spot of stone trees, bitter rot
12 of June 2016	garden soap K + methylated spirits	15 l/ha + 15 l/ha	aphids
19 of June 2016	garden soap K + methylated spirits	15 l/ha + 15 l/ha	aphids
27 of June 2016	garden soap K + methylated spirits	15 l/ha + 15 l/ha	aphids
8 of July 2016	garden soap K + methylated spirits	15 l/ha + 15 l/ha	aphids
17 of July 2016	garden soap K + methylated spirits	15 l/ha + 15 l/ha	aphids
22 of July 2016	garden soap K + methylated spirits	15 l/ha + 15 l/ha	aphids
8 of August 2016	garden soap K + methylated spirits	15 l/ha + 15 l/ha	aphids
3 of March 2017	Cuprate Extra 350 SC	3 l/ha	brown rot, leaf spot of stone trees, bitter rot
30 of March 2017	Cuprate Extra 350 SC	1.5 l/ha	brown rot, leaf spot of stone trees, bitter rot
4 of August 2017	Cuprate Extra 350 SC	1.5 l/ha	brown rot, leaf spot of stone trees, bitter rot
26 of March 2018	Cuprate Extra 350 SC	1,5 l/ha	brown rot, leaf spot of stone trees, bitter rot
12 of April 2018	Cuprate Extra 350 SC	1,5 l/ha	brown rot, leaf spot of stone trees, bitter rot
20 of April 2018	Efective Microorganisms (Farma Plus)	20 l/ha	brown rot, leaf spot of stone trees, bitter rot, plant feeding
21 of May 2018	Cuprate Extra 350 SC	1,5 kg/ha	brown rot, leaf spot of stone trees, bitter rot
5 of December 2018	Cuprate Extra 350 SC	1,5 kg/ha	brown rot, leaf spot of stone trees, bitter rot

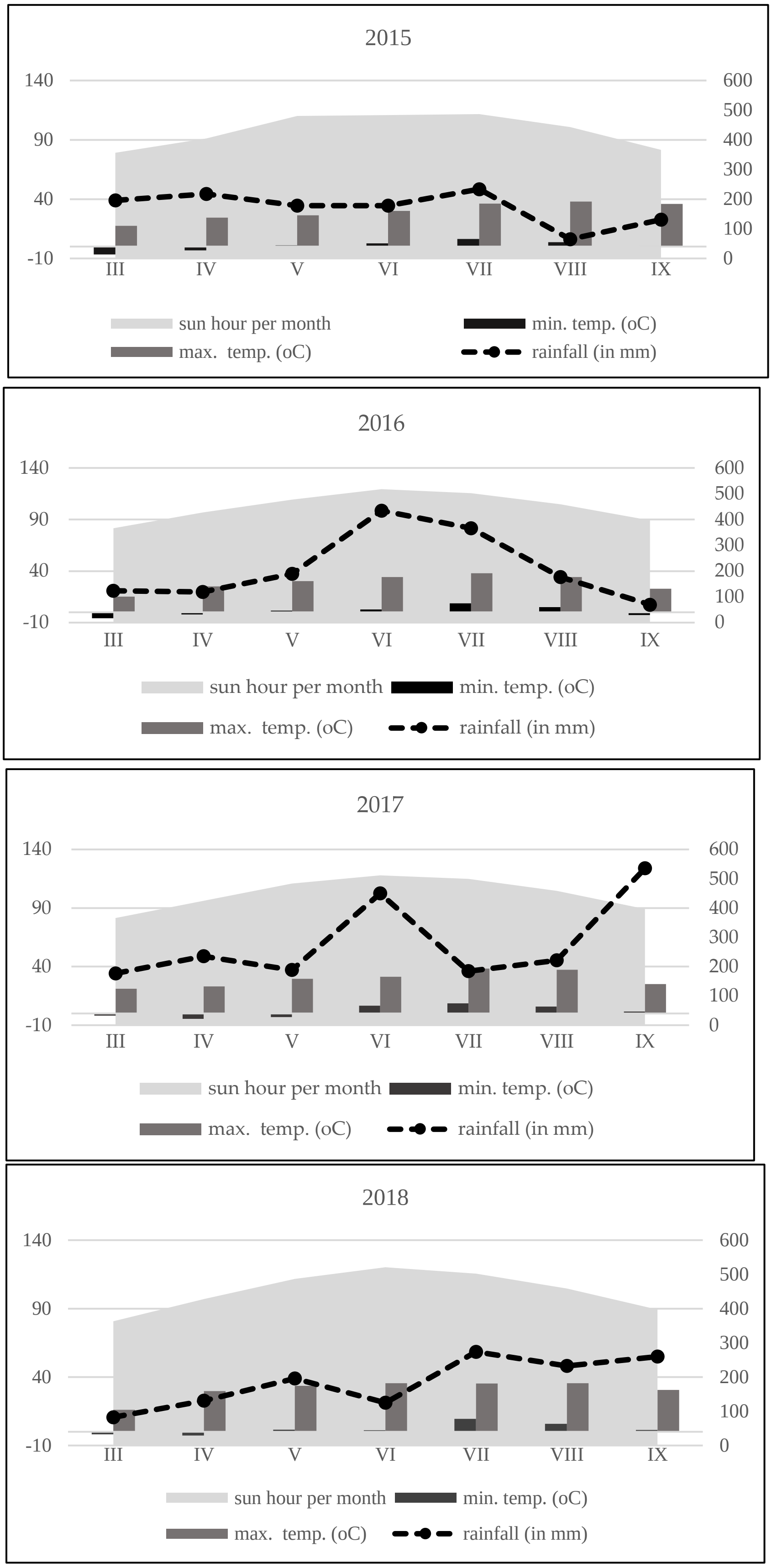

Figure S1. Weather condition in four year of experiment with sour cherry cultivars in organic and conventional orchards
